# The Activities and Impact of State Programs to Address Hereditary Breast and Ovarian Cancer, 2011–2014

**DOI:** 10.3390/healthcare3040948

**Published:** 2015-10-15

**Authors:** Katrina F. Trivers, Juan L. Rodriguez, Summer L. Cox, Barbara E. Crane, Debra Duquette

**Affiliations:** 1 Division of Cancer Prevention and Control, National Center for Chronic Disease Prevention and Health Promotion, Centers for Disease Control and Prevention, Atlanta, GA 30341, USA; E-Mail: fph4@cdc.gov; 2 Oregon Health Authority, Portland, OR 97232, USA; E-Mail: summer.l.cox@state.or.us; 3 Georgia Department of Public Health, Atlanta, GA 30303-3142, USA; E-Mail: Barbara.Crane@dph.ga.gov; 4 Michigan Department of Health and Human Services, Lansing, MI 48909, USA; E-Mail: DuquetteD@michigan.gov

**Keywords:** genomics, genetic services, implementation, state health departments, BRCA, breast cancer, ovarian cancer, hereditary breast, ovarian cancer

## Abstract

In 2011, the Division of Cancer Prevention and Control (DCPC), at the United States Centers for Disease Control and Prevention (CDC), released a three-year funding opportunity announcement (FOA) for a competitive, non-research cooperative agreement. The agreement enhanced the capacities of state health departments to promote the application of best practices for evidence-based breast cancer genomics through education, surveillance, and policy activities. The FOA required that applicants focus on activities related to hereditary breast and ovarian cancer (HBOC). The DCPC funded three states: Georgia, Michigan, and Oregon. Georgia was a first-time recipient of cancer genomics funding, whereas Michigan and Oregon had long standing activities in cancer genomics and had received CDC funding in the past. By the end of the funding period, each state had well-functioning and impactful state-based programs in breast cancer genomics. This article highlights the impact of a few key state activities by using CDC’s Science Impact Framework. There were challenges to implementing public health genomics programs, including the need to develop relevant partnerships, the highly technical nature of the subject matter, a lack of genetic services in certain areas, and the difficulty in funding genetic services. Georgia, Michigan, and Oregon have served as models for others interested in initiating or expanding cancer genomics programs, and they helped to determine what works well for promoting and integrating public health genomics into existing systems.

## 1. Introduction

Breast Cancer Susceptibility gene (BRCA) mutations are estimated to occur in 1 in 300 to 500 women in the general population [1,2,3,4]. BRCA mutation carriers have up to a 65% risk of developing female breast cancer by age 70 years [5,6] and up to a 39% risk in developing ovarian, fallopian tube, or peritoneal cancer by age 70 years [5,6]. Interventions among mutation carriers and other high-risk women, including prophylactic surgery, can reduce breast cancer risk by 85% to 100% [7,8,9] and ovarian cancer risk by 69% to 100% [10]. In order to reduce their risk of cancer, mutation carriers first need to be identified and, second, they need to undergo clinical interventions. Although there are evidence-based clinical recommendations on hereditary breast and ovarian cancer risk assessment, genetic counseling, and genetic testing in the United States [11,12], there are few evidence-based programs that promote or implement these clinical recommendations at a population level. Since 2005, the United States Preventive Services Task Force (USPSTF) has recommended that women at high risk of developing Hereditary Breast and Ovarian Cancer (HBOC) should be referred for genetic counseling and evaluation for genetic testing. In addition, women at low risk of developing HBOC should not be referred for such services [11,13]. Despite these recommendations, there are few coordinated public health cancer genomics programs in the United States. The lack of scalable and effective public health programs for the prevention of hereditary cancers is an important gap and highlights the need to turn evidence-based guidelines into practice to improve the population’s health [14]. In this article, we describe the activities and impact of CDC-funded programs for breast and ovarian cancer genomics.

## 2. Experimental Section and Methods

To address this gap, in 2011, the Division of Cancer Prevention and Control (DCPC) at the Centers for Disease Control and Prevention (CDC) released a funding opportunity announcement (FOA) for a competitive, nonresearch cooperative agreement, Enhancing Breast Cancer Genomic Practices Through Education, Surveillance, and Policy. This FOA was intended to enhance the capacities of state health departments to promote best practices for evidence-based breast cancer genomics by using education, surveillance, and policy. Policy and education programs are needed to facilitate the translation of evidence-based genomic applications into practice, to help develop proper evidence for using genomic applications, and to aid health professionals and the public to understand the potential harms and benefits of evidence-based breast cancer genomic applications. In addition, surveillance is needed about the positive and negative uses and outcomes of breast cancer genomic applications.

The overall purpose of the cooperative agreement, which was modeled after those previously released by CDC’s Office of Public Health Genomics (OPHG) [15], was to promote the adoption of evidence-based recommendations about HBOC into public health practice, consistent with the Healthy People 2020 objectives in the focus areas of Genomics and Cancer: Increase the proportion of women with a family history of breast and/or ovarian cancer who receive genetic counseling (G-1); Reduce the overall cancer death rate (C-1); Reduce the female breast cancer death rate (C-3); Reduce late-stage female breast cancer (C-11) [16].

The FOA required that state health departments conduct policy or systems change activities and either surveillance or education activities. States could also propose to conduct activities in all three areas. The FOA did not require states to undertake specific activities within those three overarching areas, since there are a variety of ways to implement these activities, based on state needs, strengths, and limitations. The funding could not be used for research, fundraising, or clinical services. Application eligibility was limited to state health departments or their bona fide agents. DCPC funded three state health departments—the Michigan Department of Health and Human Services (Michigan), the Oregon Health Authority (Oregon), and the Georgia Department of Public Health (Georgia)—for a three-year period, and each grantee received approximately $ 300,000 per year. Georgia was a first-time recipient of cancer genomics funding and had minimal cancer genomics activities before funding, whereas Michigan and Oregon had long standing activities in cancer genomics and had received CDC funding in the past.

Authorization and funding for this cooperative agreement was partially given by a provision of the Patient Protection and Affordable Care Act (Section 10413, Part V, Section 399 NN of the Public Health Service Act, “Young Women’s Breast Health Awareness and Support of Young Women Diagnosed with Breast Cancer”) [17]. This provision authorized the CDC to conduct activities related to breast cancer in young women.

## 3. Results and Discussion

### 3.1. Broad Overview of State Activities

All three grantees proposed education, surveillance, and policy activities in accordance with the FOA and consistent with state-based priorities. Grantees developed or enhanced activities for the promotion of breast cancer genomics. Activities were designed to increase appropriate BRCA 1/2 counseling and testing, increased insurance coverage of BRCA 1/2 and related clinical interventions for appropriate women, and included the development of educational programs to further the public’s and health care providers’ knowledge about family history, risk assessment, and the appropriateness of BRCA1/2 counseling and testing. Next, we briefly describe examples of state-specific activities.

Educational activities were directed at the public and health care providers. Health care providers were educated by using in-person conferences, online modules and webinars, and provider newsletters. Both Georgia and Oregon, with CDC and other partners, collaborated with Michigan to create an online educational module for health care providers, which addressed the assessment of cancer family history for genetic risk and appropriate referral to genetic counseling and testing. All grantees included promotion and dissemination of this module in their educational efforts. Patient education activities conducted by the grantees included the development of web pages, lectures, newsletters, displays at health fairs, and information provided at clinician’s offices. Some of these public health activities were intended for high-risk populations, such as young breast cancer survivors and individuals of Ashkenazi-Jewish decent.

Many of the surveillance activities used existing infrastructure, such as adding questions to the state’s annual Behavioral Risk Factor and Surveillance Survey (BRFSS) and analyzing state cancer registry data. Michigan and Oregon also used state cancer registry data to identify individuals with cancer diagnoses indicative of possible HBOC, and reported those cases back to the provider and cancer survivor, if possible, along with educational information about genetic counseling and testing (a process known as bi-directional cancer registry reporting). In addition, new surveillance systems were developed with key partners and stakeholders to assess uptake of HBOC genetic counseling, testing, and follow-up services. Michigan and Oregon developed new cancer genetic counseling and testing surveillance programs by partnering with genetics clinics across their respective states to collect data about patients accessing HBOC counseling services, including testing outcomes and follow-up services. Georgia conducted a survey of breast cancer survivors in their state to assess the uptake of genetic counseling, and the state tested and surveyed first and third-year primary care medical residents about their knowledge of HBOC and genetic counseling and testing recommendations. Georgia also collaborated with nine public health clinics across the state to integrate a genetic cancer risk-screening tool into their clinical intake process.

Policy and systems change activities across all three states were fairly similar. All programs worked with health insurance companies, including private and, where possible, public options in their states, to ensure that coverage policies were consistent with evidence-based recommendations for referral to genetic counseling and testing (e.g., the USPSTF or National Comprehensive Cancer Network^®^, (NCCN^®^, Washington, PA, USA) Clinical Practice Guidelines In Oncology (NCCN Guidelines^®^, Washington, PA, USA)). This work was accomplished in a variety of ways depending on the state, but included meeting with health insurance plan medical directors, conducting key informant interviews and focus groups with health plan administrators to assess barriers and facilitators to having evidence-based coverage policies, and developing and disseminating policy guidance documents for insurers. Each state worked collaboratively with their state comprehensive cancer control (CCC) program; genomics objectives and activities were newly incorporated into Georgia’s CCC plans.

Grantees also worked with governmental entities to improve cancer genomics practice within their respective states. For instance, Oregon worked with partners and provided educational materials about the importance of licensure for genetic counseling. As in many states, genetic counselors are not licensed in Oregon, thus creating barriers for use of services. Georgia, through the state Breast Cancer License Plate Program, created a fund to cover costs of genetic testing for at-risk, underserved women. Michigan disseminated, at no cost, educational brochures with model informed consent forms, as aligned with a state law that requires written informed consent to be obtained by the ordering health care provider before presymptomatic or predictive genetic testing.

#### Impact of Selected, Key State Activities

To describe outcomes of the cancer genomics FOA, the DCPC traced the impact of key state education, surveillance, and policy activities by using CDC’s science impact framework [18] illustrative examples from each state are presented here (Table 1). This is the first time that this framework has been used to show the impact of state cancer genomic activities.

**Table 1:** Example state activities addressing hereditary breast and ovarian cancer, 2011–2014.

State	Activity	Brief Description of Activity	Type of Activity: Education, Surveillance, or Policy
Michigan Department of Health and Human Services (Michigan)	Identification of educational needs followed by development and dissemination of a free online educational module with CMEs	Michigan identified health care provider knowledge gaps by using surveys and other data sources. A free, online, educational module was then developed with attached Continuing Medical Education credits (CMEs) called, *Hereditary Breast and Ovarian Cancer: Is Your Patient at High Risk?*	Education
	Honoring health insurance plans for having evidence-based genomic services policies	Michigan reviewed health insurance company policies on *BRCA* counseling, testing, and related clinical services and gave nonmonetary awards to health plans for having written policies consistent with evidence-based recommendations. They also held focus groups with eight health plan administrators to understand and address barriers and facilitators to the uptake of evidence-based policy by health plans.	Policy
Georgia Department of Public Health (Georgia)	Incorporating a hereditary cancer risk assessment tool into clinical practice	Georgia incorporated a risk assessment tool, the Breast Cancer Genetics Referral Screening Tool (B-RST), into clinical practice in 9 of 18 public health districts across the state. The screening tool quickly identified women seen at these public health clinics who were appropriate for referral to genetic counseling. Before the incorporation of the tool in the nine health centers, an educational program was provided for all clinical and clerical staff who provided services to women.	Surveillance and Education
Oregon Health Authority (Oregon)	Tracking and promotion of genomics services in the Oregon Medicaid program.	Oregon worked closely with its state Medicaid program to track and promote use of evidence-based genomic tests.	Surveillance and Policy
	Bidirectional reporting between the cancer registry and cancer survivors and physicians.	Oregon implemented bidirectional reporting (*i.e*., cancer survivors who were likely to be appropriate for *BRCA* counseling were identified, and they and their doctors were notified and received educational materials through the Oregon State Cancer Registry).	Education and Surveillance

The science impact framework is used for tracking CDC science and linking its influence or impact on subsequent events and actions that ultimately lead to improving health. The framework is meant to be used in a variety of disciplines and can be used in other settings. It can be used to track the impact of any body of scientific work including, but not limited to, a manuscript from a research study, the development of a public health guideline, or work resulting from a grant application, and can be applied to domestic and international programs which address other health conditions. The framework is an adaptation and extension of the Institute of Medicine (IOM) Degrees of Impact framework [19] and was developed in 2012 by a workgroup of CDC scientists. Rather than relying on traditional measures of impact or dissemination (e.g., bibliometrics), the science impact framework takes the broader societal, environmental, cultural, and economic value into consideration and uses narrative, quantitative, and qualitative indicators. Because impact often takes a long time to become apparent, the focus is on uncovering short-term indicators that are indicative of long term impact. 

**Figure 1:**
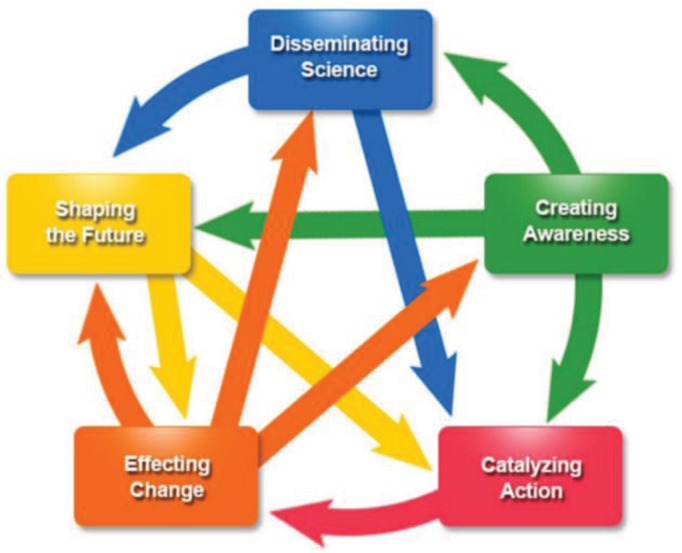
The science impact framework [18].

The framework (Figure 1, Table 2) is a series of five domains of scientific influence that define degrees of impact and may not be chronological; events do not have to happen in every domain, and the degree of impact is not a progression (feedback at all levels is possible). The five domains are Disseminating Science, Creating Awareness, Catalyzing Action, Effecting Change, and Shaping the Future. Disseminating Science includes generating and communicating knowledge by the producer. Key indicators for this include publications of findings in the peer-reviewed literature, presentations at conferences or meetings, and other dissemination efforts. Creating Awareness represents the uptake of knowledge and further dissemination and dialogue by the user, and acceptance of a concept or findings by others, including those in the scientific or public health community or policy makers. Key indicators include receipt of awards, stakeholder-created resources, curriculum or training, receipt of feedback from others through surveys, focus groups, anecdotes, information sharing, and communications among professional societies, or receipt of queries from outside groups. Catalyzing Action reflects the adoption of knowledge resulting in specific actions. Indicators could include partnerships and collaborations, technology creation, or introduction into public health or clinical practice. Effecting Change includes changes to current or existing situations, directions, strategies, policies, or practice. Indicators could include building public health capacity; legal or policy change; cultural, social, or behavioral change; or economic change. Shaping the Future is characterized by the implementation of new activities based on the initial activity or furthering improvements and changes. Key indicators could include new hypotheses, continuous quality improvement, or the implementation of public health programs or initiatives. The science impact framework is a framework emphasizing influence and categorization of activities into the various domains is a bit arbitrary; one activity could possibly be placed in a variety of categories. We placed activities in the one domain they best fit into based on the intent of the activity. Ultimately, it is less important in which domain individual activities are categorized than describing how activities are linked together.

**Table 2:** The domains of influence within the science impact framework and example indicators (modified from [18]).

Domain of Influence	Potential Measureable Indicators
Disseminating Science: Generating and communicating knowledge by the producer	Scientific publications (open access journals) Trade publications Professional meetings/conferences General communication (social media, web, print) Presentations Training, coursework Other scientific output (e.g., CDC Public Health Grand Rounds, Vital Signs, Science Clips)
Creating Awareness: The uptake of knowledge and further dissemination and dialogue by the user, and acceptance of a concept or findings by others	Continuing Education (CME, CEU) Awards Stakeholder resources, curriculum, training Feedback (Survey, focus groups, anecdote) Information sharing and communications among professional societies Electronic communications (information shared on listservs and other electronic resources, social media, news coverage) Queries Requests to contribute to efforts that further the science output
Catalyzing Action: Adoption of knowledge resulting in specific actions	Technology creation New funding (pilots/research) Advocacy groups/NGOs Congressional hearings Partnerships and collaborations Research & Development Office practice/point of care changes
Effecting Change: Changes in current or existing situations, directions, strategies, policies, or practice	Building public health capacity (e.g., workforce development, funded research, improved staff competency) Creation of registries/surveillance Legal/policy changes Accreditation Cultural/social change Behavioral change Economic change CMS reimbursement Other payer actions Change instilled (New) formal guidelines and recommendations (e.g., WHO) Hospital standards Funding Anecdotes/case studies Sustainable and scalable science translation
Shaping the Future: Implementing new or furthering improvements and changes	New hypotheses/Continuous Quality Improvement Implementation of public health programs/initiatives Health outcomes Prevalence and incidence Morbidity and mortality (e.g., frequency of outbreaks, trends) Life expectancy Quality of Life improvements Reductions in economic burden

### 3.2. Michigan Department of Health and Human Services (Michigan) Activities

#### 3.2.1. Education: Identification of Educational Need Followed by Development and Dissemination of a Free Online Educational Module with CMEs 

Michigan identified health care provider knowledge gaps by using surveys and other data sources. As one of their key educational activities, a free, online, educational module was developed with attached Continuing Medical Education credits (CMEs) called, *Hereditary Breast and Ovarian Cancer: Is Your Patient at High Risk?* The development and impact of this activity can be traced as follows.

##### Disseminating Science

Michigan developed and implemented a health care provider survey, and results were used to identify knowledge gaps and plan targeted education. Results from the survey showed limited knowledge of basic information about hereditary cancer, management and prevention strategies, and limited use of family history data to determine who to refer for genetic services. For example, only one-third of health care providers correctly identified autosomal dominant as the most common mode of inheritance for most hereditary cancer syndromes; less than one-half collected cancer family history when deciding who to refer to cancer genetics; and less than one-third correctly identified prophylactic oophorectomy as the procedure that most reduces risk of cancer for females aged 40 years with known *BRCA* mutations [20]

Michigan developed the online CME module, Hereditary Breast and Ovarian Cancer: Is Your Patient at Risk? [21], with Jackson Laboratory and other partners, (including the Georgia and Oregon grantees), in part on the basis of the results of the survey and data from other sources. This free CME course was launched January 2014. The CME is currently available online with CMEs available through October 2016, and was widely promoted through formal and informal channels, including CDC’s National Breast and Cervical Cancer Early Detection Program (NBCCEDP) grantees, local health care providers, health insurance companies in Michigan, the MedEdPortal, a DCPC blog post, and an OPHG weekly update e-mail.

##### Creating Awareness

As of February 2015, the site had received many page views (9500 page views from 1800 users in 2200 sessions), but there was limited uptake of CMEs (59 people took the post-test to get CMEs, and 40 passed). Most who took the test for CME credit were from Michigan, and an evaluation is ongoing, the results of which will be used to improve the training and dissemination plans. 

##### Catalyzing Action

Michigan collaborated with many clinical partners, state organizations, health systems, health plans, advocacy and non-profit groups to promote several educational tools, including the online CME module.

##### Shaping the Future

Discussions are underway for how to integrate genomics into existing cancer prevention programs funded by DCPC, and how best to leverage expertise and collaborate across diverse programs (*i.e*., comprehensive cancer control, cancer surveillance, and cancer screening). For example, the DCPC recently developed and hosted a webinar about genomics and risk assessment for the NBCCEDP, given reimbursement changes (*i.e.*, the program will now reimburse for MRIs for certain high-risk women without cancer). This work also informs key DCPC priorities and initiatives (e.g., Inside Knowledge: Get the Facts about Gynecologic cancer [22], and work related to Breast Cancer in Young Women [23]). The promotion of already created educational materials, including the online CME module, will be a part of many of those activities.

#### 3.2.2. Policy: Honoring Health Insurance Plans for Having Evidence-Based Genomic Services Policies 

##### Catalyzing Action

Partnerships with insurance companies may increase the likelihood such companies have written evidence-based policies for their members. As one of their key policy activities, Michigan reviewed health insurance company policies on *BRCA* counseling, testing, and related clinical services and gave nonmonetary awards to health plans for having written policies consistent with evidence-based recommendations. Michigan also held focus groups with eight health plan administrators to understand and address barriers and facilitators to the uptake of evidence-based policy by health plans. Michigan also partnered with a state health plan professional organization and sought input from 15 health plan medical directors about *BRCA* counseling, testing, and clinical services policies.

##### Disseminating Science

Along with the awards, Michigan also developed and distributed newsletters and packets to health plans to educate administrators about the importance and cost-effectiveness of these services. These packets included the USPSTF *BRCA* Grade B and Grade D Recommendations, informational flyers about the *Hereditary Breast and Ovarian Cancer (HBOC): Is Your Patient at Risk?* CME online module, samples of exemplary health plan policies for *BRCA* Clinical Services, relevant clinical guidelines, the Michigan Informed Consent Law Brochure with Model Consent Form, and additional resources from state and national groups.

##### Effecting Change

Because of Michigan’s educational and policy work with health plans, the state was able to document improvements in coverage for genetic services. There were increases in the number of health plans with written evidence-based policies (from 4 out of 25 in 2008 to 16 out of 25 in 2014) and there was a decrease in reports of inadequate insurance coverage as a barrier to testing (23.8% in 2009 to 11.1% in 2013 as ascertained through their clinical database of those seeking BRCA-related counseling in cancer genetics clinics). For these efforts, Michigan also was honored with the 2014 Spirit of Collaboration award from their state cancer consortium.

### 3.3. Georgia Department of Public Health (Georgia) Activities

#### 3.3.1. Surveillance: Incorporating a Hereditary Cancer Risk Assessment Tool into Clinical Practice

##### Catalyzing Action

As one of their key surveillance and education activities, Georgia incorporated a risk assessment tool, the Breast Cancer Genetics Referral Screening Tool (B-RST), into clinical practice in 9 of 18 public health districts across the state. Each district contains one or more health centers. All of the centers that incorporated the B-RST provided family planning and breast and cervical cancer screening services to low-income, uninsured, and underserved women. Each of nine sites were selected in an attempt to reach high-risk and underserved populations (e.g., those of Ashkenazi Jewish descent and African-Americans).

The B-RST was developed before the cooperative agreement by a key collaborator of the Georgia Department of Public Health [24,25]. The use of the B-RST is included in the United States Preventive Services Task Force 2013 *BRCA-related Cancer: Risk Assessment, Genetic Counseling and Genetic Testing* grade B recommendation. This recommendation describes the B-RST as, “one of several screening tools designed to identify a family history that may be associated with an increased risk for potentially harmful mutations in breast cancer susceptibility genes (*BRCA1* or *BRCA2*)” [11]. The screening tool, which can be accessed at https://www.breastcancergenescreen.org/ [26], quickly identified women seen at these public health clinics who were appropriate for referral to genetic counseling. Before the incorporation of the tool in the nine health centers, an educational program was provided for all clinical and clerical staff who provided services to women. This educational program included a lecture about HBOC and the introduction of a guidance manual that contained the clinical care and follow-up algorithms, copies of educational materials available for the woman, and an explanation of the screening tool (B-RST) and the website. Use of the tool was also demonstrated.

##### Effecting Change

Because of the incorporation of B-RST into the clinics, 3636 women were screened by the end of the three-year funding period; 213 (5.9%) had a positive screen and were referred for genetic services; 60% of those with a positive screen (n = 128) were able to be contacted. Among those who received genetic services (n = 127), the 30 people who were appropriate for BRCA testing received testing (one was positive for a mutation, two had variants of unknown significance and 27 were negative). Funding for genetic testing was not directly available through these public health clinics; therefore alternative sources of funding had to be identified. Myriad provided free genetic testing for low-income and uninsured women. Underinsured or Medicaid-insured women received testing from a fund developed from the sale of the Georgia Breast Cancer license tag. Georgia’s experience highlights the interest and ability of clinics to incorporate genetic risk assessment tools into busy practices, including those who serve traditionally underserved populations.

##### Disseminating Science

An article was published describing the implementation of the B-RST and initial results from the screening [27]. All three grantees actively disseminate information about the B-RST when communicating with providers, highlighting it as an example of an easy to use tool that could be readily and easily incorporated into primary health care practices.

### 3.4. Oregon Health Authority (Oregon) Activities

#### 3.4.1. Surveillance/Policy: Tracking and Promotion of Genomics Services in the Oregon Medicaid Program

Oregon developed partnerships with its state Medicaid program (Oregon Health Plan) to promote use of evidence-based genomic tests in multiple ways. The impact of this work can be traced as follows:

- Creating Awareness. Starting in 2011, a key collaborator of the Oregon Health Authority chaired and facilitated the Genetic Advisory Council (GAC) to the Health Evidence Review Commission (HERC). The HERC provides coverage guidance and sets priorities for health spending in the Oregon Health Plan and promotes evidence-based medical practice statewide. Oregon and the GAC worked closely to create guidelines and a decision-making algorithm for nonprenatal genetic testing, thus creating awareness among key stakeholders and reflecting acceptance of the concept that genetic services are important. Oregon also worked closely with GAC and the HERC Value-based Benefits Subcommittee to review updated molecular pathology CPT codes and recommended coverage of appropriate tests to ensure that coverage priority was set by using the most current evidence-based genetic information.
- Disseminating Science. Oregon conducted and disseminated results of analyses of Medicaid claims data annually to the Oregon Medicaid program (*i.e.*, to the HERC, the medical director, and managed care directors). These results showed increases in the number of *BRCA* tests ordered, from 6.7 per 100,000 persons in 2008, to 33.2 per 100,000 persons in 2012, but usage was still lower than expected on the basis of the population covered by Medicaid, indicating underutilization. However, they were unable to determine appropriateness of testing given the incomplete personal and family history data available. These data are paramount as Oregon is one of the few states in which Medicaid covers genetic testing, and these data were used to track progress towards improving coverage for these services.
- Creating Awareness. Because of the dissemination of the claims data, ongoing discussions between Oregon and the Oregon Health Plan ensued. On the basis of feedback from the Medicaid program and their review of the results, they concluded that the low usage in the program was likely caused by a lack of knowledge about coverage for these services.
- Catalyzing Action. Because of the lack of knowledge about coverage for these services, Oregon developed a form for clinicians to use when ordering genetic tests for Medicaid clients to aid in coverage decisions by managed care directors. In addition, Oregon added information about Medicaid coverage for genetics in their educational materials in 2012. Medicaid’s health plan policy, which includes coverage of genetic counseling and testing for *BRCA* based on USPSTF and NCCN Guidelines^®^, Washington, PA, USA, was implemented in 2011 and has been updated regularly. Collaborations between the two programs continue.

#### 3.4.2. Education: Bidirectional Reporting between the Cancer Registry and Cancer Survivors and Physicians

##### Catalyzing Action

As one of their key educational and surveillance activities, Oregon implemented bidirectional reporting at the state level. In this case, bidirectional reporting was a process by which cancer survivors who were likely to be appropriate for *BRCA* counseling were identified, and they and their doctors were notified and received educational materials through the Oregon State Cancer Registry. Oregon identified living patients who were diagnosed with ovarian cancer, breast cancer at a young age (≤ 50 years), and male breast cancer; deceased cancer patients were removed via links with vital records data. These groups of patients were selected because of their increased likelihood of having a hereditable cancer syndrome, which made them likely to be appropriate candidates for referral to genetic counseling and, if indicated after counseling, genetic testing. 

##### Disseminating Science

In 2013, Oregon developed and disseminated targeted public and provider educational materials to 2,801 cancer survivors and 1,253 health care providers and asked them to complete a survivor or provider survey. Results from this study have been presented at state, national, and international conferences [28,29]. Results were also shared at a local provider training event and with national stakeholders, among other disseminations.

##### Effecting Change

Because of bidirectional reporting and receipt of education materials, 40% of the cancer survivors who had never received genetic counseling (n = 193) and 47% of cancer survivors who had never received testing (n = 153) reported that a letter prompted them to take action, such as seeing a genetics expert or speaking with family members. Oregon also attempted to determine the impact of bidirectional reporting on health care providers’ behavior. A survey was distributed, but low response rates precluded analyses.

#### 3.4.3. Other Noteworthy Impacts of the FOA not Directly Linked to above

##### Effecting Change

In addition to those specifically mentioned before, other impacts related to the FOA have occurred. Because of the CDC funding, Georgia now has state-wide breast cancer genomic activities where none existed before 2011, including the continuation and expansion of incorporating the B-RST into their breast and cervical cancer screening program and family planning programs. In addition, Congress has seen value in the work conducted through the FOA and encouraged the CDC to expand its cancer genomics efforts. In CDC’s FY 2015 appropriations bill, the state-based cooperative agreements were specifically mentioned in appropriations report language: “T*he Committee understands that ovarian cancer is expected to claim the lives of more than 14,000 women this year, and there is no test to help identify the disease early when it is most treatable and the chance of survival is greatest. The Committee is pleased that CDC has undertaken pilot efforts in three States to promote breast/ovarian cancer genomics best practices designed to educate women and providers about the BRCA mutation, identify women at high risk, and help ensure appropriate referral for genetic counseling or testing. The Committee encourages CDC to expand these efforts.”* [30].

##### Shaping the Future

CDC released a new FOA and round of cancer genomics funding in 2014, which expanded the number of years of funding (from three to five), the number of grantees (three to four), funding amount ($ 300,000 per grantee per year to approximately $ 350,000 per grantee per year), and scope (not just hereditary breast and ovarian cancer to both hereditary breast and ovarian cancer and Lynch Syndrome, if desired). Discussions are underway as to how to integrate genomics into existing programs funded by DCPC, how best to leverage expertise and collaborate across our diverse national programs (e.g., comprehensive cancer control, cancer surveillance, cancer screening).

## 4. Conclusions

The science impact framework is a valuable tool to determine the effect of CDC programmatic funding. By the end of the three-year funding period, all three states had well-functioning and effective state-based programs in breast cancer genomics that included innovative education, surveillance, and policy activities. These states serve as models for others interested in initiating or expanding cancer genomics programs, and their novel activities provide data to determine what works well in the promotion of public health genomics. Public health genomics programs are unique because they require specific and sometimes highly technical expertise in genomics, as well as expertise in the basics of program management and key public health activities (e.g., surveillance, education). Because of this unique nature, developing strong collaborations and partnerships is key to the success of this work, including collaborations with other CDC-funded programs and with external partners (e.g., clinical medical centers, nonprofits). Other challenges to the implementation of public health genomics include the lack of genetic services in certain areas and the difficulty in funding genetic services. However, despite ongoing challenges, promising activities could be implemented in other states and nationwide. Even public health institutions without dedicated genomics funding could implement some activities, including those described in CDC’s genomic applications toolkit for public health departments [31]. Expanding this work has the ability to decrease disease and death associated with hereditary cancers by means of primary prevention, through clinical preventive interventions, and early detection. 
